# Soft tissue sarcoma subtypes exhibit distinct patterns of acquired uniparental disomy

**DOI:** 10.1186/1755-8794-5-60

**Published:** 2012-12-05

**Authors:** Musaffe Tuna, Zhenlin Ju, Christopher I Amos, Gordon B Mills

**Affiliations:** 1 Department of Epidemiology, Unit 1340, The University of Texas MD Anderson Cancer Center, 1515 Holcombe Blvd, Houston, TX 77030-4009, USA; 2 Department of Bioinformatics and Computational Biology, The University of Texas MD Anderson Cancer Center, Houston, TX, USA; 3 Department of Genetics, The University of Texas MD Anderson Cancer Center, Houston, TX, USA; 4 Department of Systems Biology, The University of Texas MD Anderson Cancer Center, Houston, TX, USA

**Keywords:** Acquired uniparental disomy, Soft tissue sarcoma and whole-genome

## Abstract

**Background:**

Soft tissue sarcomas (STS) are heterogeneous mesenchymal tumors with diverse subtypes. STS can be classified into two main categories according to the type of genomic alteration: recurrent translocation driven STS, and non-recurrent translocations. However, little has known about acquired uniparental disomy in STS.

**Methods:**

In this study, we analyzed SNP microarray data to determine the frequency and distribution patterns of acquired uniparental disomy (aUPD) in major soft tissue sarcoma (STS) subtypes using CNAG and R softwares.

**Results:**

We identified recurrent aUPD regions specific to alveolar rhabdomyosarcoma with the most frequent at 11p15.4, gastrointestinal stromal tumor at 1p36.11-p35.3, leiomyosarcoma at 17p13.3-p13.1, myxofibrosarcoma at 1p35.1-p34.2 and 16q23.3-q24.1, and pleomorphic liposarcoma at 13q13.2-q13.3 and 13q14.11-q14.2. In contrast, specific recurrent aUPD regions were not identified in dedifferentiated liposarcoma, Ewing sarcoma, myxoid/round cell liposarcoma, and synovial sarcoma. Strikingly total, centromeric and segmental aUPD regions are more frequent in STS that do not exhibit recurrent translocation events.

**Conclusions:**

Our study yields a detailed map of aUPD across 9 diverse STS subtypes and suggests the potential location of several novel tumor suppressor genes and oncogenes.

## Background

Soft tissue sarcoma (STS) is a heterogeneous disease with 50 clinically relevant subtypes with different histology, molecular genetic profiles, tumor locations, and prognosis [1-3]. Genomic alterations including specific DNA copy number alterations, [4] chromosomal translocations, and mutations are hallmarks of different subtypes of STS. STS can be classified into two main categories according to the type of genomic alteration: i) recurrent translocation driven STS, with reciprocal translocation resulting in oncogenic fusion transcripts (e.g. *EWSR1-FLI1* in Ewing sarcoma, *SS18-SSX* in synovial sarcoma, *PAX3-FOXO1* in alveolar rhabdomyosarcoma (aRMS), *FUS-CHOP* in myxoid/round-cell (MRC) liposarcoma), and [5] ii) STS with non-recurrent translocations (e.g. myxofibrosarcoma, leiomyosarcoma, liposarcoma [dedifferentiated liposarcoma and pleomorphic liposarcoma]). The non-recurrent translocation group tends to show complex genomic changes including gains/amplifications and deletions in multiple chromosomal regions, [3,6] or activating mutations (e.g. *KIT* and *PDGFRA*) in gastrointestinal stromal tumors (GIST). According to the World Health Organization (WHO) classification, liposarcomas are further classified into four morphological subtypes: well-differentiated liposarcoma, de-differentiated liposarcoma, pleomorphic liposarcoma, and myxoid/round-cell liposarcoma (MRC), which enables characterization of the individual liposarcoma subtypes [7]. *MDM2* amplification at chromosome 12q13-q15, which is present in all tumor samples, is a key driver of dedifferentiated liposarcoma [8,9]. Pleomorphic liposarcoma tumor samples harbor gains and deletions in multiple chromosomal regions with the most common being deletion (60%) at chromosome 13q14.2-q14.3 (*RB1*) in addition to complex genomic rearrangements [10]. Mutations are common in different types of liposarcoma including *TP53* mutations in 17% of pleomorphic liposarcomas; *NF1* mutations in 10.5% of myxofibrosarcoma, 8% of pleomorphic liposarcomas; and *PIK3CA* mutations in 18% of MRC [11].

Single nucleotide polymorphism (SNP) microarrays allow the detection of copy number alterations and acquired uniparental disomy (aUPD also known as copy number neutral loss of heterozygosity), which occurs when both copies of a chromosome originate from the same parent, in the most cases without a change in copy number. There are two major mechanisms leading to aUPD: mitotic recombination of sister chromatids, which results to segmental aUPD, or the loss of a complete chromosome followed by duplication resulting in whole chromosome aUPD.

aUPD regions may cause pre-existing abnormalities (mutation, deletion, amplification, methylation, histone modification, and/or imprinting) to become homozygous, which may lead to clonal selection and growth advantage in the cells. To date, aUPD has been described mostly in hematologic malignancies, [12-15] breast cancer [16-18] and colon cancer [19]. Barretina and colleagues have recently reported aUPD in a limited number of STS samples [11]. The purpose of this study was to determine the frequency, distribution of aUPD in 9 subtypes of STS and identify recurrent aUPD regions specific for each subtype in a large sample set of STS.

## Methods

We retrieved raw data (Affymetrix GeneChip Human DNA-oligonucleotide SNP array CEL files) from the Gene Expression Omnibus (GEO) database (http://www.ncbi.nlm.nih.gov/geo) for a total of 319 soft tissue sarcoma (STS) tumor samples from 5 GEO series with the following accession numbers: GSE8046 (20 samples), [20] GSE15696 (10 samples), GSE20709 (25 samples), [21] GSE21124 (207 tumor samples), [11] and GSE24715 (57 samples) (Additional file 1: Table S1) [22,23]. We included 315 STS tumor samples that passed quality control (QC) to determine distribution of genome-wide aUPD pattern, and excluded 4 samples due to fail the quality control. The 315 samples consisted of the following subtypes: alveolar RMS (57), EWS (10), GIST (45), leiomyosarcoma (27), liposarcoma (115 samples), of which 24 were pleomorphic liposarcoma, 21 MRC liposarcoma, and 50 were dedifferentiated liposarcoma, or liposarcoma without subclassified (20 samples), myxofibrosarcoma (38), and synovial sarcoma (23).

After quality control of the retrieved raw data (CEL files), we processed the CEL (intensity) files to generate CHP files by using GeneChip Genotyping Analysis (GTYPE, version 4.1) and Genotyping Console (GTC, version 3.0) software (Affymetrix, Santa Clara, CA). QC metrics was calculated as default in GTC. Then, microarray data were analyzed for determination of allele-specific copy numbers using CNAG (Copy number analysis for GeneChips) (version 3.4) software (http://genome.umin.jp) by using a Hidden Markov Model to predict the presence of aUPD regions as previously described [24]. Data from each of the array platforms were independently analyzed by using non self-controls with automatically selected sex-matched reference samples from HapMap data and from previously published, publicly available datasets; GSE14860 [25], GSE10922 [26], GSE11417 [27], GSE10092 [28], and GSE15097 [29]. Only GSE21124 data set was analyzed by matching normal samples. In the aUPD analyses both the genotype information and the intensity were used. Then all the data from each array were used to generate aUPD profiles for each tumor. The total aUPD was calculated by counting the all aUPD regions. The segmental aUPD was calculated by counting the aUPD at telomeric and centromeric regions, and whole chromosomal aUPD was considered if aUPD occurs in entire chromosome. If aUPD occurs with one mitotic recombination defined as telomeric, and if aUPD occurs via two or more mitotic recombination defined as centromeric. The NCBI Build 36/hg18 (http://genome.ucsc.edu) was used for identifying gene localization and function. Previously, aUPD was detected in limited number of STS by using GISTIC analysis, which is designed to identify copy number alterations, but not aUPD [11].

### Statistical analyses

We performed non-parametric Kruskal-Wallis test to identify difference of frequency of aUPD regions between translocation and non-translocation groups of STS and aUPD regions between segmental and whole chromosome, telomeric and centromeric. Frequency of aUPD describes the number of aUPD per sample. Percentage of aUPD in each of groups or subtypes was calculated by the tumors that had at least one aUPD region. A two-sided *p* value < 0.05 was considered to be statistically significant. Statistical analysis was performed using R software version 2.15.0 (http://www.r-project.org/).

## Results and discussion

### Distribution of aUPD patterns in STS

**Figure 1:**
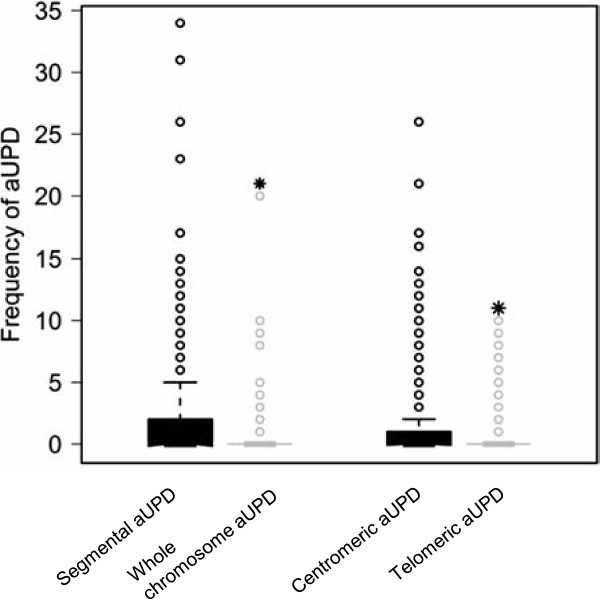
Frequency of segmental, whole chromosome, centromeric and telomeric aUPD in all samples.

We integrated genomic data from 5 different studies to allow us to interrogate a large number of samples encompassing different types of STS. As indicated in Additional file 2: Figure S1, aUPD is found across all chromosomes in STS. We identified aUPD in 47.9% (151/315) of tumor samples with a range between 0 and 37 regions (mean 2.3, median 0) with a total of 724 aUPD regions. **S**egmental aUPD (630/724; mean 2, median 0) was more frequent (*P* < 2.3E-16) than whole-chromosome aUPD (94/724; mean 0.3, median 0) (Figure 1), suggesting that mitotic recombination is a more common mechanism of aUPD generation in STS than is the loss of one chromosome and duplication of the remaining chromosome. In addition, we found that centromeric aUPD (441/724; mean 1.4, median 0) was significantly more common (*P* < 0.0002) than telomeric aUPD (189/724; mean 0.6, median 0) (Figure 1). This requires complex chromosomal rearrangements indicating that multiple mitotic recombination events occur frequently in soft tissue sarcoma tumorigenesis.

**Figure 2:**
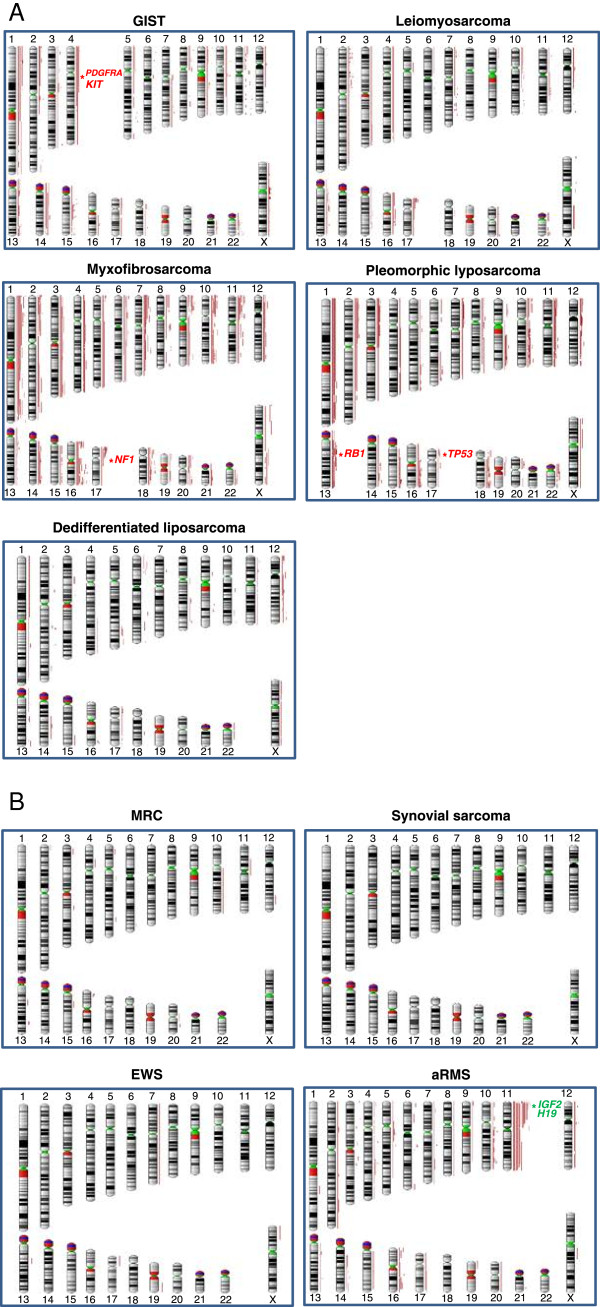
**Distribution of aUPD in (A) non-translocation and (B) translocation driven soft tissue sarcomas. **(**A**) aUPD regions in non-translocation driven soft tissue sarcomas; GIST, leiomyosarcoma, myxofibrosarcoma, pleomorphic liposarcoma, and dedifferentiated liposarcoma. (**B**) aUPD regions in translocation driven soft tissue sarcomas; myxoid/round cell liposarcoma, synovial sarcoma, Ewing sarcoma, and alveolar rhabdomyosarcoma. Each red line represents region of aUPD for each soft tissue sarcoma sample. Gene name in red represents most mutated genes and green represents imprinted genes that previously reported, which are mapped in the aUPD regions.

**Figure 3:**
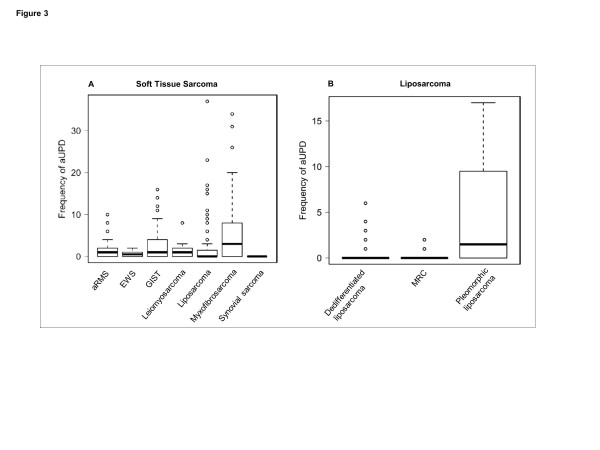
Frequency of total aUPD in (A) each subtype of soft tissue sarcomas; aRMS, EWS, GIST, leiomyosarcoma, liposarcoma, myxofibrosarcoma and synovial sarcoma, and (B) each subgroup of liposarcoma; dedifferentiated liposarcoma, pleomorphic liposarcoma and myxoid/round cell liposarcoma.

**Figure 4:**
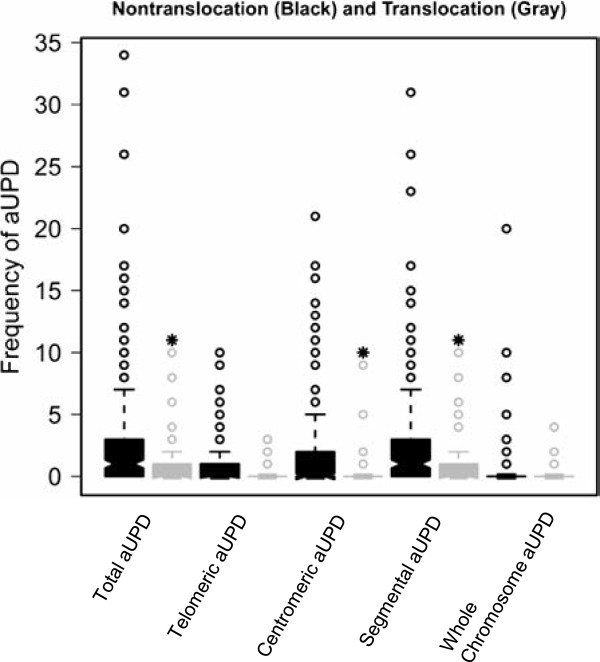
The comparison of frequency of total, telomeric, centromic, segmental and whole chromosome aUPD in non-translocation and translocation driven soft tissue sarcomas.

Strikingly, the patterns of aUPD varied markedly across STS subtypes (Figure 2A and 2B). Moreover, the proportion of patients with aUPD were found to vary in each subtype; 73% (27/37) in myxofibrosarcoma, 62.2% (28/45) in GIST, 61.4% (35/57) in alveolar rhabdomyosarcoma, 51.9% (14/27) in leiomyosarcoma, 50% (5/10) in EWS, 35.7% (41/115) in liposarcoma, and no aUPD in synovial sarcoma (0/23) (Additional file 3: Figure S2A), and the frequencies which are the counts of aUPD in each tumor sample are significantly different among these subtypes (P<2.61E-08, Figure 3A). The proportions of patients with aUPD are also different among the three subgroups of liposarcoma: 70.8% (17/24) in pleomorphic liposarcoma, 24.0% (12/50) dedifferentiated liposarcoma, and 14.3% (3/21) MRC liposarcoma (Additional file 3: Figure S2B). The frequencies of aUPD of these three subgroups are significantly different (P<9.43E-06, Figure 3B). Total, centromeric and segmental aUPD were significantly more frequent in non-recurrent translocation STS than recurrent translocation driven STS (*P* < 3.71E-04, *P* < 6.64E-06, *P* < 1.57E-04, respectively) (Figure 4). We also identified statistically significant differences in total, centromeric and segmental aUPD between subtypes of liposarcoma when comparing pleomorphic liposarcoma to MRC (*P* < 9.26E-05, *P* < 6.23E-04, *P* < 2.12E-04, respectively), and also comparing both pleomorphic liposarcoma and dedifferentiated liposarcoma to MRC (*P* < 0.02, *P* < 0.03, *P* < 0.03). However, no statistically significant difference was observed between dedifferentiated liposarcoma and MRC (*P* < 0.28, *P* < 0.26, *P* < 0.35). The frequencies of total, telomeric, centromeric, segmental and whole-chromosome aUPD for each subtype of STS are summarized in Additional file 4: Figure S3 A-E.

### Recurrent aUPD regions

We then assessed the frequency of specific aUPD regions across different STS subtypes (Figure 2A and 2B). Overall, the frequency of aUPD across all STS was highest at chromosome 11p (11.7%) and lowest at 19q (1.1%). Specific recurrent aUPD regions were found in non-recurrent translocation driven STS; GIST (Figure 2A), leiomyosarcoma (Figure 2A), myxofibrosarcoma (Figure 2A), and pleomorphic liposarcoma (Figure 2A), but not in recurrent translocation driven sarcomas; MRC (Figure 2B), synovial sarcoma (Figure 2B), and EWS (Figure 2B), with the exception of dedifferentiated liposarcoma (Figure 2A) and alveolar RMS (Figure 2B).

In GIST, the most frequent aUPD region at chromosome 1p36.11-p35.3 (15.2%) harbors candidate cancer genes including *FGR*, *RCC1*, and *TAF12*, with less frequent aberrations at chromosome 14q11.2-q21.3 (11.1%), at 4q (8.9%) where *KIT* and *PDGFRA* are located, and at chromosome 22q11.22-q12.1 (6.7%) (*SMARCB1*, *GSTT1*, *GSTT2* and *MYO18B*) (Figure 2A, Additional file 5: Table S2). Mutations of *KIT* (75%) and *PDGFRA* (28%), [21,30,31] and deletions at chromosome 14q (68–70%), 1p (53–56%), and 22q (40%) are common in GIST [11,21,32]. Genes at chromosome 1p (*KIF1B, UBE4B, PRDM2* and *TP73*), and at chromosome 14q (*RTN1, DAAM1* and *DACT1*) reported to be under-expressed in GIST samples, are found in regions of aUPD [21]. Gunawan et al. reported three cytogenetic pathways proposed to lead to the initiation and progression of GIST; one initiated with deletion at 14q, another initiated with deletion at 1p, and the last one initiated with deletion at 22q [32]. GISTs with deletion at chromosome 14q were associated with better disease free survival (DFS) (*P* < 0.005), whereas tumors with deletion at chromosome 1p (*P* < 0.00007) and 22q (*P* < 0.004) were associated with poorer DFS [32]. Taken together, our data indicates that not just gain-of-function mutations like *KIT* and *PDGFRA* but also loss-of-function mutations and reduction to homozygosity through aUPD at chromosomes 1p, 14q and 22q may contribute to pathophysiology of GIST.

In leiomyosarcoma, in contrast, the most frequent aUPD region was at chromosome 17p13.3-p13.1 (*TP53*) (25.9%) (Figure 2A, Additional file 5: Table S2), whereas in myxofibrosarcoma the most frequent aUPD was at chromosome 1p35.1-p34.2 (*EIF2C4* and *EIF2C3*) (26.3%) (Figure 2A, Additional file 5: Table S2). Copy number analysis studies have shown that deletion at chromosome 17p is common in leiomyosarcoma tumor samples, [33] indicating that the 17p region may harbor tumor suppressor genes that may be homozygously mutated or methylated following aUPD.

In pleomorphic liposarcoma, another non-translocation-related sarcoma, we found aUPD region at 13q (*TNFSF11* and *RB1*) with the most frequent aUPD at chromosome 13q13.2-q13.3 (*SMAD9*) (Figure 2A, Additional file 1: Table S1). Previously deletion [10] and aUPD [11] in the *RB1* region at chromosome 13q14.2 were reported in pleomorphic liposarcoma. Thus, aUPD at 13q13.2-q13.3 and 13q14.11-q14.2 regions may render cells homozygous of novel genes for existing abnormalities.

In alveolar RMS, the most frequent aUPD region was at chromosome 11p (29.8%), with the minimal recurrent region at 11p15.4. Several potential cancer genes map to this region: *TAF10*, *ILK*, and *EIF3F* (Figure 2B, Additional file 5: Table S2). aRMS is characterized by loss of imprinting in *IGF2* and *H19*[34-36]. Interestingly IGF2 is expressed from the paternal allele, which may lead to increased expression of *IGF2* while H19 is maternal expressed, and may lead to suppressed expression of *H19*[37]. Thus aUPD in these regions could result in decreased or increased expression of candidate cancer genes depending on which parental allele is duplicated.

### Homozygous deletion and focal amplification at aUPD regions

Next, we identified aUPD regions with homozygous deletion or focal amplification. aUPD regions with homozygous deletion that could potentially harbor tumor suppressor genes are summarized in Additional file 6: Table S3. We found a focal amplification region at chromosome 11p15.3-p15.2 (*RASSF10*, *RRAS2*, and *COPB1*) in one tumor sample of aRMS, where the other aRMS samples harbor aUPD in the same region (Additional file 6: Table S3). The amplification at 11p15.3-p15.2 may increase the level of a gain-of-function allele in this region.

## Conclusion

In conclusion, to our knowledge, our study encompasses the largest sample set available for the analysis of aUPD in soft tissue sarcoma subtypes. Our results yield a detailed map of aUPD across 9 diverse sarcoma subtypes. The frequency and distribution of aUPD is significantly higher in fusion-negative STS than translocation driven STS suggesting an alternative mechanism underlying tumor development. This study provides evidence for a basis for mutation screening with next-generation sequencing to identify potential mechanistic mediators and therapeutic targets for each subtype of STS and particularly for recurrent regions specifically associated with translocation negative STS.

## Abbreviations

aUPD: acquired uniparental disomy; EWS: Ewing sarcoma; GEO: Gene Expression Omnibus; GIST: Gastrointestinal stromal tumor; MRC: Myxoid/round cell liposarcoma; RMS: Rhabdomyosarcoma; SNP: Single nucleotide polymorphism.

## Competing interests

The authors declare no conflict of interest.

## Authors’ contributions

MT and GBM designed the study. MT and ZJ analysed data. MT, GBM, ZJ and CIA interpreted results. MT drafted manuscript. MT, GBM, ZJ and CIA made critical revision to manuscript. All authors have read and approved the final manuscript.

## Pre-publication history

The pre-publication history for this paper can be accessed here:

http://www.biomedcentral.com/1755-8794/5/60/prepub

## Supplementary Material

Additional file 1 **Table S1. **SNP microarray data summary. Click here for file

Additional file 2 **Figure S1. **Distribution of aUPD regions in all soft tissue sarcoma samples. Each brown line represents aUPD region in each sample. Click here for file

Additional file 3 **Figure S2. **The percentage of aUPD in (A) each subtype of STS and (B) each subgroup of liposarcoma. Click here for file

Additional file 4 **Figure S3. **The frequency of aUPD in translocation and non-translocation driven soft tissue sarcomas. The frequency of (A) total aUPD, (B) telomeric aUPD, (C) centromeric aUPD, (D) segmental aUPD, (E) whole chromosome aUPD in non-translocation and translocation driven tumors. Click here for file

Additional file 5 **Table S2. **Properties of recurrent aUPD regions in tumor samples of aRMS, GIST, leiomyosarcoma, myxofibrosarcoma, and pleomorphic liposarcoma tumor samples Click here for file

Additional file 6 **Table S3. **aUPD regions with homozygous deletions and focal amplifications in tumor samples of soft tissue sarcoma subtypes. Click here for file
