# Supplementary material for: Soft tissue sarcoma subtypes exhibit distinct patterns of acquired uniparental disomy
Source: BMC Med Genomics. 2012 Dec 5;5:60. doi: 10.1186/1755-8794-5-60 (PMC3541987; doi:10.1186/1755-8794-5-60)
Supplement: Additional file 1 — Table S1. SNP microarray data summary. [file 1755-8794-5-60-S1.doc]

**Supplementary Table 1**. SNP microarray data summary.

| **Data**  **set** | **GEO accession number** | **Number of samples** | **STS subtypes** | **Platforms**  **(Affymetrix)** |
| --- | --- | --- | --- | --- |
| 1. | GSE8046 | 20 | Liposarcoma | 50K Xba |
| 2. | GSE15696 | 10 | EWS | 250K Nsp |
| 3. | GSE20709 | 23* | GIST | SNP 6.0 |
| 4. | GSE21124 | 205* | Leiomyosarcoma  GIST  Myxofibrosarcoma  Pleomorphic liposarcoma  Dedifferentiated liposarcoma  Myxoid/round-cell liposarcoma (MRC)  Synovial sarcoma | 250K StyI |
| 5. | GSE24715 | 57 | Alveolar Rhabdomyosarcoma | 50K Xba |

* Two samples from each set were excluded due to fail the quality control.
