# Supplementary figures and images for: Soft tissue sarcoma subtypes exhibit distinct patterns of acquired uniparental disomy
Source: BMC Med Genomics. 2012 Dec 5;5:60. doi: 10.1186/1755-8794-5-60 (PMC3541987; doi:10.1186/1755-8794-5-60)

## Slide 1
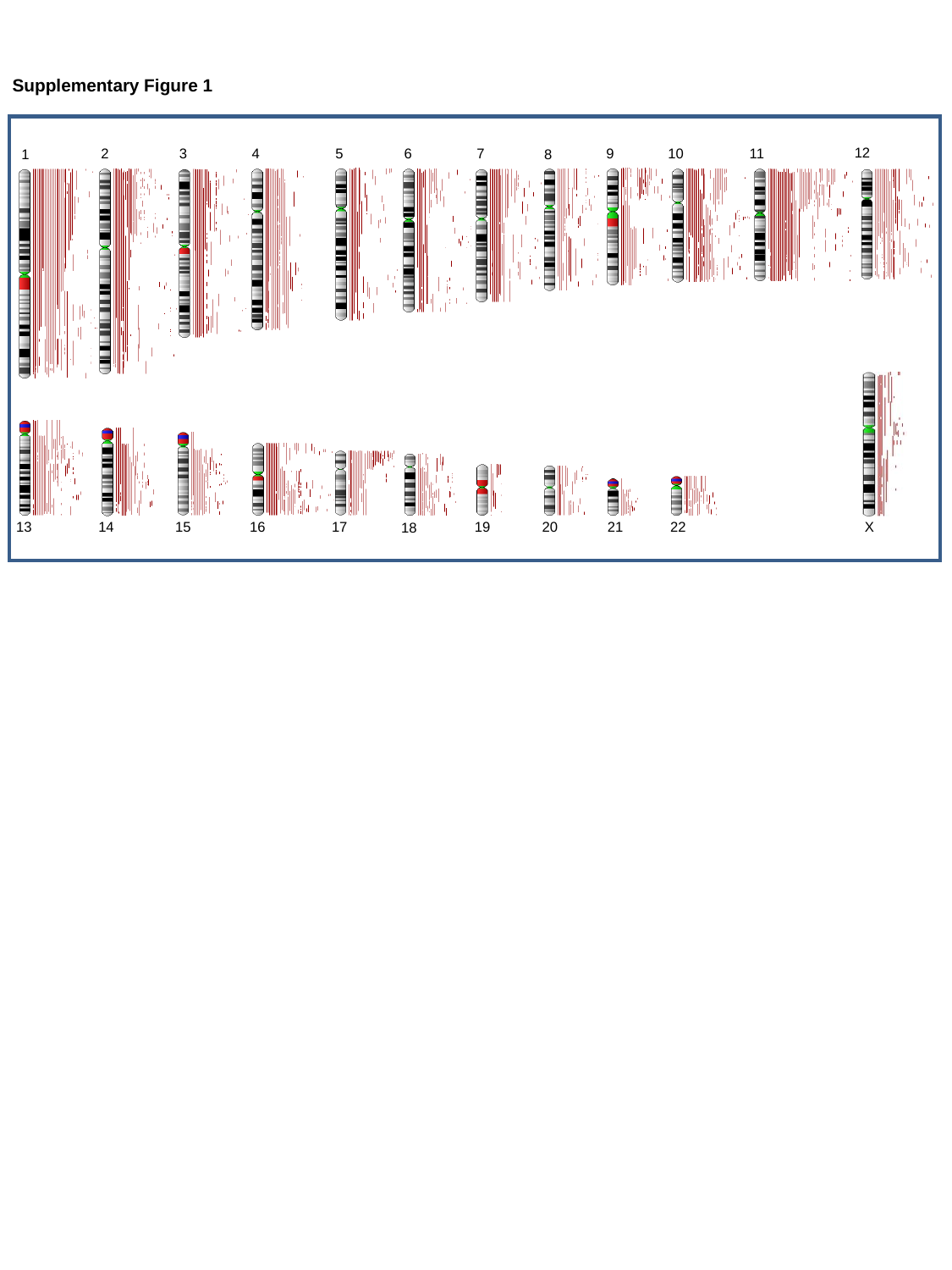

Supplementary Figure 1
12
9
10
11
2
7
3
4
5
6
1
8
17
13
15
19
20
21
16
X
14
22
18

Supplement: Additional file 2 — Figure S1. Distribution of aUPD regions in all soft tissue sarcoma samples. Each brown line represents aUPD region in each sample. [file 1755-8794-5-60-S2.ppt]

## Slide 1
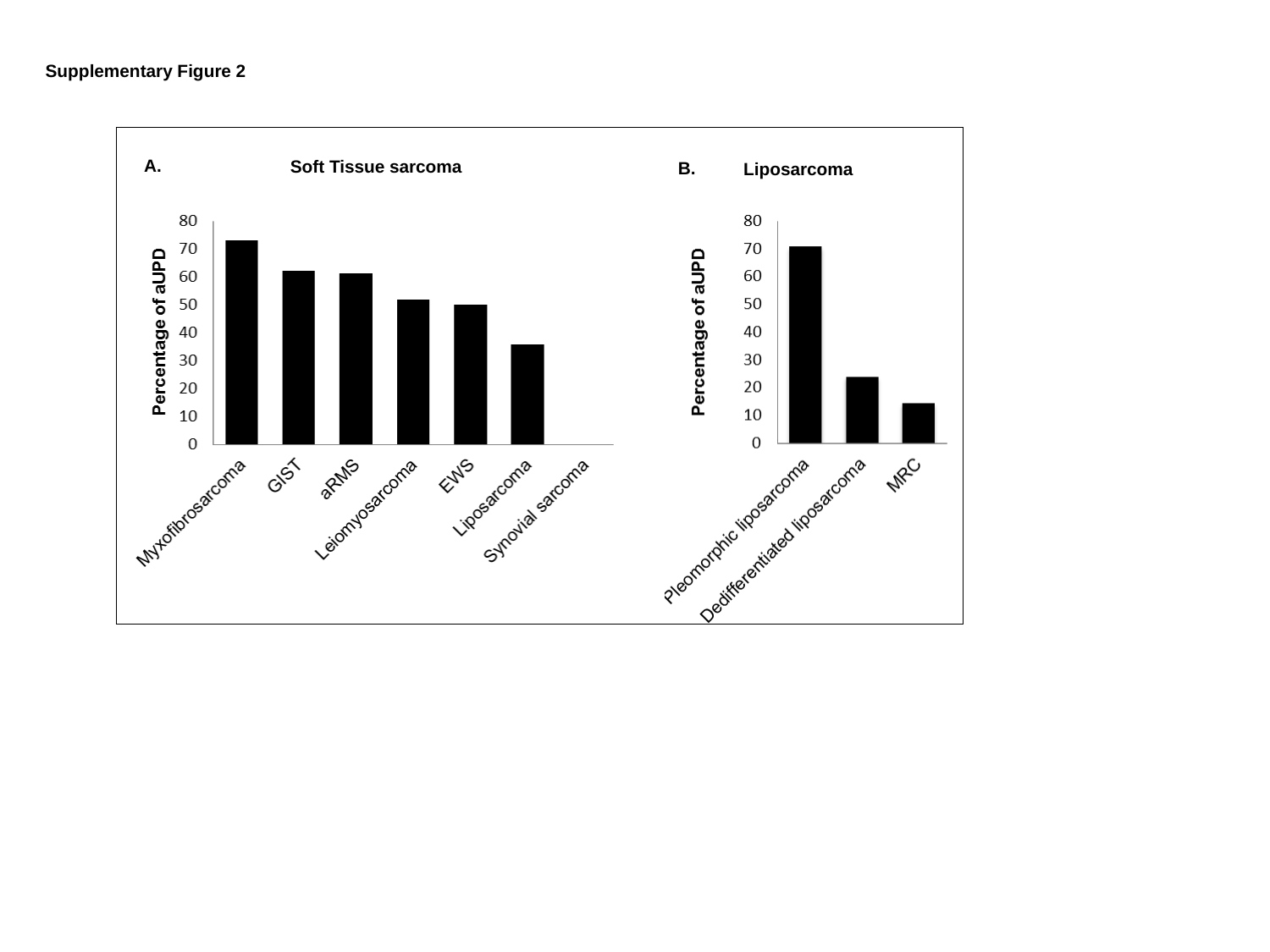

Supplementary Figure 2
A.
Soft Tissue sarcoma
B.
Liposarcoma

Supplement: Additional file 3 — Figure S2. The percentage of aUPD in (A) each subtype of STS and (B) each subgroup of liposarcoma. [file 1755-8794-5-60-S3.ppt]
