# Supplementary material for: Soft tissue sarcoma subtypes exhibit distinct patterns of acquired uniparental disomy
Source: BMC Med Genomics. 2012 Dec 5;5:60. doi: 10.1186/1755-8794-5-60 (PMC3541987; doi:10.1186/1755-8794-5-60)
Supplement: Additional file 4 — Figure S3. The frequency of aUPD in translocation and non-translocation driven soft tissue sarcomas. The frequency of (A) total aUPD, (B) telomeric aUPD, (C) centromeric aUPD, (D) segmental aUPD, (E) whole chromosome aUPD in non-translocation and translocation driven tumors. [file 1755-8794-5-60-S4.ppt]

## Slide 1
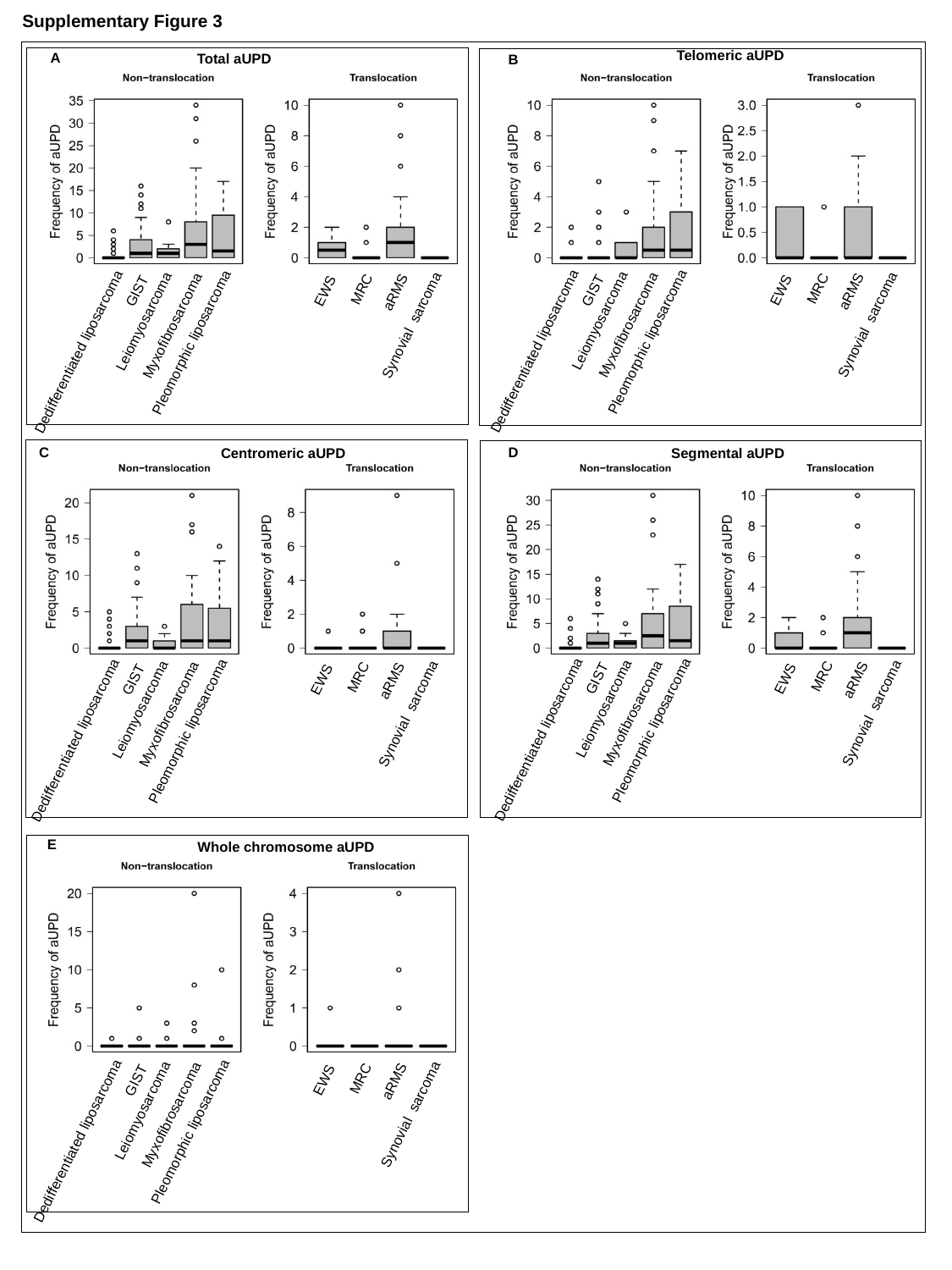

Supplementary Figure 3
Telomeric aUPD
A
Total aUPD
B
MRC
GIST
EWS
aRMS
Leiomyosarcoma
Synovial sarcoma
Myxofibrosarcoma
Pleomorphic liposarcoma
Dedifferentiated liposarcoma
MRC
GIST
EWS
aRMS
Leiomyosarcoma
Synovial sarcoma
Myxofibrosarcoma
Pleomorphic liposarcoma
Dedifferentiated liposarcoma
D
C
Segmental aUPD
Centromeric aUPD
MRC
GIST
EWS
aRMS
Leiomyosarcoma
Synovial sarcoma
Myxofibrosarcoma
Pleomorphic liposarcoma
Dedifferentiated liposarcoma
MRC
GIST
EWS
aRMS
Leiomyosarcoma
Synovial sarcoma
Myxofibrosarcoma
Pleomorphic liposarcoma
Dedifferentiated liposarcoma
E
Whole chromosome aUPD
MRC
GIST
EWS
aRMS
Leiomyosarcoma
Synovial sarcoma
Myxofibrosarcoma
Pleomorphic liposarcoma
Dedifferentiated liposarcoma
