# Supplementary material for: Soft tissue sarcoma subtypes exhibit distinct patterns of acquired uniparental disomy
Source: BMC Med Genomics. 2012 Dec 5;5:60. doi: 10.1186/1755-8794-5-60 (PMC3541987; doi:10.1186/1755-8794-5-60)
Supplement: Additional file 5 — Table S2. Properties of recurrent aUPD regions in tumor samples of aRMS, GIST, leiomyosarcoma, myxofibrosarcoma, and pleomorphic liposarcoma tumor samples [file 1755-8794-5-60-S5.doc]

**Supplementary Table 2. Properties of recurrent aUPD regions in tumor samples of aRMS, GIST, leiomyosarcoma, myxofibrosarcoma, and pleomorphic liposarcoma tumor samples**

| **STS subtype** | **Chromosomal region** | **Start point** | **End point** | **Length**  **(bp)** | **Frequency**  **(%)** | **Some genes in the region** |
| --- | --- | --- | --- | --- | --- | --- |
| ***Alveolar RMS*** | | | | | | |
|  | 11p15.4 | 5,324,847 | 7,987,270 | 2,662,424 | 17/57 (29.8) | *EIF3F, TAF10, ILK* |
| ***GIST*** | | | | | | |
|  | 1p36.11-p35.3 | 26,520,640 | 29,559,464 | 3,038,824 | 7/45 (15.6) | *FGR*, *RCC1*, *EPB41*, *RAB42*, *TAF12* |
|  | 4p16.1 | 8,883,509 | 10,549,167 | 1,665,659 | 4/45 (8.9) | *USP17*, *WDR1* |
|  | 10q21.1 | 58,336,744 | 59,174,113 | 837,370 | 6/45 (13.3) | None |
|  | 14q11.2-q21.3 | 19,645,914 | 46,588,881 | 26,942,967 | 5/45 (11.1) | *BCL2L2, NFKBIA, PAX9, FOXA1* |
|  | 14q23.3-q24.1 | 65,392,827 | 69,041,354 | 3,648,527 | 5/45 (11.1) | *RAD51L1* |
|  | 15q.1 | 38,613,569 | 40,304,675 | 1,691,107 | 5/45 (11.1) | *RAD51*, *TMEM87* |
| ***Leiomyosarcoma*** | | | | | | |
|  | 2p24.1-p23.3 | 21,213,198 | 24,014,941 | 2,801,744 | 4/27 (14.8) | *ATAD2B* |
|  | 13q21.1-q21.2 | 56,490,314 | 59,279,959 | 2,789,646 | 4/27 (14.8) | *PCDH17* |
|  | 13q21.31-q21.32 | 61,604,663 | 64,161,838 | 2,557,175 | 4/27 (14.8) | None |
|  | 16q12.1-q12.2 | 50,155,762 | 51,327,625 | 1,171,864 | 4/27 (14.8) | *TOX3* |
|  | 16q12.2-q13 | 53,436,980 | 54,608,843 | 1,171,863 | 4/27 (14.8) | *CRNDE* |
|  | 16q21 | 59,530,670 | 61,171,279 | 1,640,610 | 4/27 (14.8) | *CDH8* |
|  | 17p13.3-p13.1 | 3,056,930 | 7,759,900 | 4,702,971 | 7/27 (25.9) | *P2RX5*, *SLC25A11*, *TP53* |
| ***Myxofibrosarcoma*** | | | | | | |
|  | 1p36.33 | 0 | 1,838,288 | 1,838,288 | 9/38 (23.7) | *HES4*, *SDF4*, *CDK11a*, *CDK11B* |
|  | 1p36.13-p36.11 | 16,774,,377 | 24,127,528 | 7,353,152 | 9/38 (23.7) | *EIF4G3*, *WNT4*, *E2F2*, *PAX7* |
|  | 1p35.1-p34.2 | 33,548,754 | 39,982,761 | 6,434,008 | 10/38 (26.3) | *EIF2C4*, *EIF2C3* |
|  | 1p32.3-p32.1 | 54,689,064 | 59,744,356 | 5,055,293 | 9/38 (23.7) | *DAB1* |
|  | 2p23.1 | 30,799,862 | 31,719,261 | 919,400 | 6/38 (15.8) | *EHD3* |
|  | 2p22.3 | 34,017,758 | 35,626,706 | 1,608,948 | 6/38 (15.8) | None |
|  | 3q26.31-q28 | 174,823,569 | 191,890,778 | 17,067,209 | 6/38 (15.8) | *PIKC3CA, BCL6, TP63* |
|  | 6p25.2-p25.1 | 3,464,189 | 6,928,378 | 3,464,190 | 6/38 (15.8) | *LYRM4* |
|  | 7p15.3-p15.2 | 22,655,748 | 25,892,284 | 3,236,537 | 7/38 (18.4) | *IGF2BP3*, *STK31* |
|  | 9p24.3-p24.1 | 1,622,996 | 5,796,415 | 4,173,420 | 6/38 (15.8) | *RFX3*, *JAK2* |
|  | 10p11.23-p11.21 | 29,671,176 | 35,929,940 | 6,258,765 | 7/38 (18.4) | *MAP3K8*, *MTPAP*, *ITGB1* |
|  | 10q25.3-q26.11 | 117,757,479 | 121,234,570 | 3,477,092 | 7/38 (18.4) | *CASC2*, *EIF3A* |
|  | 10q26.12-q26.13 | 122,857,213 | 124,248,049 | 1,390,837 | 7/38 (18.4) | *FGFR2*, *ATE1* |
|  | 10q26.13 | 124,711,661 | 127,261,528 | 2,549,868 | 7/38 (18.4) | *BUB3* |
|  | 11p15.1-p14.3 | 19,240,599 | 21,790,558 | 2,549,960 | 7/38 (18.4) | *DBX1* |
|  | 16q21-q22.1 | 62,577,516 | 65,389,988 | 2,812,473 | 8/38 (21.1) | *CDH11*, *CDH5* |
|  | 16q23.3-q24.1 | 81,796,078 | 83,671,060 | 1,874,983 | 10/38 (26.3) | *CDH13* |
|  | 17p13.3-p13.2 | 2,116,336 | 3,762,376 | 1,646,041 | 7/38 (18.4) | *TSR1* |
|  | 17p13.3-p13.1 | 3,292,079 | 7,995,048 | 4,702,971 | 8/38 (21.1) | *TP53*, *CHD3* |
|  | 17p11.2 | 18,576,730 | 19,752,472 | 1,175,743 | 7/38 (18.4) | *FBXW10*, *MAPK7* |
|  | 18q21.2-q21.31 | 47,602,676 | 53,494,096 | 5,891,421 | 7/38 (18.4) | *TCF4*, *WDR7* |
| ***Pleomorphic liposarcoma*** | | | | | | |
|  | 2p25.1-p24.1 | 11,206,972 | 22,413,945 | 11,206,973 | 6/24 (25.0) | *MYCN* |
|  | 2p22.2-p21 | 38,023,656 | 46,028,636 | 8,004,980 | 6/24 (25.0) | *EML4* |
|  | 2p14-p12 | 69,243,079 | 77,648,308 | 8,405,230 | 6/24 (25.0) | *TGFA*, *NAT8* |
|  | 10q23.31-q23.33 | 91,875,950 | 96,347,788 | 4,471,839 | 5/24 (20.8) | *CPEB3* |
|  | 13q13.2-q13.3 | 34,650,548 | 39,134,736 | 4,484,189 | 7/24 (29.2) | *SMAD9* |
|  | 13q14.11-q14.2 | 41,988,311 | 46,880,153 | 4,891,843 | 7/24 (29.2) | *TNFSF11* |
|  | 16q12.2-q13 | 53,296,356 | 55,362,106 | 2,065,751 | 6/24 (25) | *SCLC6A2* |
|  | 16q24.1 | 83,456,309 | 84,695,760 | 1,239,452 | 6/24 (25) | *COX4NB* |

Abbreviations: GIST, gastrointestinal stromal tumor; RMS, rhabdomyosarcoma.
