# Supplementary material for: Soft tissue sarcoma subtypes exhibit distinct patterns of acquired uniparental disomy
Source: BMC Med Genomics. 2012 Dec 5;5:60. doi: 10.1186/1755-8794-5-60 (PMC3541987; doi:10.1186/1755-8794-5-60)
Supplement: Additional file 6 — Table S3. aUPD regions with homozygous deletions and focal amplifications in tumor samples of soft tissue sarcoma subtypes. [file 1755-8794-5-60-S6.doc]

**Supplementary Table 3. aUPD regions with homozygous deletions and focal amplifications in tumor samples of soft tissue sarcoma subtypes.**

| **Sarcoma subtype** | **Chromosomal region** | **Start point** | **End point** | **Length**  **(bp)** | **Number of samples** | **Candidate genes** |
| --- | --- | --- | --- | --- | --- | --- |
| *Homozygous deletion* | | | | | | |
| Alveolar RMS | 11q14.1 | 83,479,618 | 84,175,464 | 714,414 | 1 | *DLG2* |
|  | 11q14.1 | 78,667,249 | 80,058,335 | 1,409,355 | 1 | *ODZ4* |
|  | 9p21.3 | 21,547,128 | 22,270,186 | 723,059 | 2 | *CDKN2A, CDKN2B* |
| Leiomyosarcoma | 7q21.11 | 77,707,944 | 77,946,660 | 238,717 | 1 | *MAGI2* |
|  | 9p24.2 | 3,800,281 | 4,070,699 | 270,419 | 1 | *GLIS3* |
|  | 10p13 | 14,479,938 | 14,681,430 | 201,493 | 1 | *No gene* |
|  | 10q22.2-q22.3 | 76,090,886 | 79,019,238 | 2,928,353 | 1 | *DUSP13* |
|  | 12p13.33 | 1,139,337 | 1,526,622 | 387,286 | 1 | *ERC1* |
|  | 13q14.2-q14.3 | 47,917,390 | 50,768,962 | 2,851,572 | 1 | *RB1, FNDC3A, TRIM13* |
|  | 17p13.3 | 6,888 | 917,847 | 910,960 | 1 | *RPH3AL* |
|  | 17p11.2-q11.1 | 21,374,237 | 22,581,677 | 1,207,440 | 1 | *FAM27L* |
| Myxofibrosarcoma | 2q37.3 | 238,212,131 | 239,162,224 | 950,094 | 1 | *TRAF3IP1* |
|  | 3p26.3 | 120,558 | 970,334 | 849,777 | 1 | *CHL1* |
|  | 3p26.1 | 7,663,662 | 7,864,900 | 201,239 | 1 | *GRM7* |
|  | 3p14.2 | 60,090,502 | 60,650,083 | 559,582 | 1 | *FHIT* |
|  | 4q12-q13.1 | 57,936,333 | 61,707,385 | 3,771,053 | 1 | *None* |
|  | 9p21.3 | 21,981,752 | 22,601,155 | 619,404 | 1 | *CDKN2A, CDKN2B* |
|  | 9q21.31 | 81,116,798 | 81,772,813 | 656,016 | 1 | *TLE4* |
|  | 12q11-q12 | 36,252,574 | 39,460,033 | 3,207,460 | 1 | *CNTN1* |
|  | 12q14.3-q15 | 65,563,129 | 66,322,984 | 759,856 | 1 | *CAND1* |
|  | 13q14.2-q14.3 | 47,917,390 | 49,974,381 | 2,056,992 | 1 | *RB1, RCBTB2, FNDC3A* |
|  | 13q21.2 | 57,652,698 | 60,160,245 | 2,507,548 | 1 | *DIAPH3* |
|  | 13q21.32 | 65,363,158 | 66,284,302 | 921,145 | 1 | *PCDH9* |
|  | 17p13.1 | 7,781,404 | 8,395,366 | 613,963 | 2 | *CNTROB* |
|  | 19p13.3 | 228,776 | 6,596,441 | 6,367,665 | 1 | *DAPK3, STK11, WDR18, NFIC* |
| MRC liposarcoma | 10q23.31 | 89,856,629 | 90,571,871 | 715,243 | 1 | *RNLS* |
| *Focal amplification* | | | | | | |
| Alveolar RMS | 11p15.3-p15.2 | 12,501,945 | 14,593,532 | 2,143,237 | 1 | *RASSF10, RRAS2, COPB1* |

Abbreviations: EWS, Ewing sarcoma; miRNA, microRNA; MRC, myxoid/round cell; alveolar RMS, alveolar rhabdomyosarcoma. A locus was determined to be homozygous deleted, if the log 2 ratio for both alleles was less that the value of the wild type (WT) reference (two copies) by at least <-0.7. Similarly, a locus was considered to be amplified if the reported sample copy number log 2 ratio was greater than the value of the WT by >1.0.
